# Assessment of the renal function and fibrosis indexes of conventional western medicine with Chinese medicine for dredging collaterals on treating renal fibrosis: A systematic review and meta-analysis

**DOI:** 10.1515/med-2023-0815

**Published:** 2024-07-17

**Authors:** Yingbo Guo, Wenfeng Gao, Xinyu Ding, Qian Cai, Yu Bai

**Affiliations:** Department of Nephropathy, Dongfang Hospital of Beijing University of Chinese Medicine, Fengtai District, 100078, Beijing, China; Department of Urology, Dongzhimen Hospital of Beijing University of Chinese Medicine, 100700, Beijing, China

**Keywords:** traditional Chinese medicine, dredging collaterals, renal fibrosis, meta-analysis

## Abstract

To investigate the renal function and fibrosis indexes of conventional western medicine with Chinese medicine for dredging collaterals in the treatment of renal fibrosis (RF). We searched articles from databases (PubMed, Embase, The Cochrane Library, CNKI, and Wanfang data) and references of included studies. The quality of literature was evaluated and data were extracted in regard to the inclusion and exclusion criteria. RevMan5.3 software was applied for all statistical analyses. Eleven eligible RCTs with a total of 898 patients were included in this meta-analysis. Compared with conventional western medicine alone, conventional western medicine with Chinese medicine for dredging collaterals in the treatment of RF has lower BUN levels and SCr levels (*P* < 0.05). As for fibrosis indexes, conventional western medicine with Chinese medicine for dredging collaterals has lower HA, laminin (LN), IV-Col, and PC-III levels (*P* < 0.05). Conventional western medicine with Chinese medicine for dredging collaterals with lower BUN, Scr, HA, LN, PC-III, and IV-Col levels, has an advantage in the treatment of RF. These lower serum levels may not be associated with the presence of RF. Ideally, kidney biopsies should be performed to confirm that these markers reduce RF. This is a major limitation of this study.

## 1 Introduction

Chronic kidney disease (CKD) refers to chronic kidney structure and function abnormalities caused by various reasons. According to statistics, the prevalence of CKD is 8–16%, and CKD has been estimated to affect more than 10% of the western population [1]. CKD has become a global public health problem due to its high morbidity and mortality [2,3]. Renal fibrosis (RF) is a common CKD feature, manifested in the abnormal and excessive deposition of extracellular matrix (ECM) protein in the glomerulus and interstitial area. Four fibrosis indexes were related to the RF biomarkers’ association with other injury-specific biomarkers: hyaluronic acid (HA), laminin (LN), type IV collagen, and procollagen III. RF is a sign of CKD progression, and the severity of CKD is usually related to the degree of RF [4]. Therefore, preventing, delay, or even reverse RF should be the key to the prevention and treatment of CKD [5]. Blockade of renin–angiotensin–aldosteronesystem (RAAS) activation is currently the only clinical treatment supported by evidence to delay the progression of RF [6]. At present, the RAAS system-blocking drugs used in clinical practice mainly include angiotensin-converting enzyme inhibitor and angiotensin II receptor blocker, both of which are the first-line recommended drugs for CKD patients with proteinuria [7,8]. A number of randomized controlled clinical trials have shown that RAAS system antagonists can effectively delay the loss of renal function and reduce the mortality of CKD patients while controlling blood pressure and urinary protein [9]. In recent years, traditional Chinese medicine used “kidney collateral syndrome” to prevent and treat RF. Studies have shown that turbid-toxin syndrome is a common witness in the later stage of kidney disease, which is mostly caused by the decay of the spleen and kidney and the internal turbid toxin. Representative prescriptions include Qihong Tongmai Drink, rhubarb worm pills, Qinghua, kidney-detoxifying Decoction, etc. [10,11,12]. It mainly used the methods of activating blood circulation and dredging the spleen and kidney collaterals. For example, Transforming growth factor-β (TGF-β) is the most closely related to the generation and accumulation of ECM. Abnormal elevation of TGF-β directly stimulates DNA synthesis and replication in renal interstitial cells. Studies have shown that TGF-β has a bidirectional regulatory effect, inhibiting inflammation and cell proliferation in the physiological state, and inducing ECM synthesis and accumulation and tissue fibrosis in the pathological state. Rhubaric acid can reverse TGF-β-induced hypertrophy of RTEC, inhibit ECM synthesis stimulated by it, and reduce RTEC regulation by down-regulating activities of NF-xB and Caspase-3. In addition, the fibrotic effect of platelet-derived growth factor (PDGF) is shown to promote the proliferation of fibroblasts, leading to an increase in the expression and synthesis of ECMs, attract inflammatory cells to chemotaxis and aggregate; cause vasoconstriction and TGF-β mutual stimulation, and promote ECM accumulation. Studies have shown that *Centella asiatica* can delay the occurrence of RF by inhibiting the high expression of PDGF and the number of fibroblasts [13,14].

There is currently no conclusion on the efficacy and safety in regard to the renal function and fibrosis indexes of conventional western medicine with Chinese medicine for dredging collaterals versus conventional western medicine alone for RF; hence, this meta-analysis aims to investigate the articles about the renal function and fibrosis indexes of using conventional western medicine with Chinese medicine for dredging collaterals in the treatment of RF.

## 2 Methods

### 2.1 Search strategy

This meta-analysis was conducted in accordance with the PRISMA 2009 guideline for systematic review and meta-analysis. Traditional Chinese herbs dredging collaterals (Tongluo Decoction formula consists of: Astragalus 30 g, Coptis chinensis 15 g, Codonopsis codonopsis 30 g, Atractylote 15 g, Poria codonopsis 15 g, angelica 15 g, Ligusticum ligusticum 10 g, forsythia forsythia 10 g, honeysuckle 15 g, Radix notoginseng 15 g, Radix spatholobi 15 g, licorice 6 g, take one dose of formula a day, add 100 mL water to decocte about 300 mL of liquid medicine. The decoction was taken after breakfast and dinner. 4 weeks as a course of treatment, continuous treatment for 3 courses), RF, CKD, randomized controlled trials (RCT) were searched in PubMed, Embase, The Cochrane Library (2020, Issue 10), and Web of Science. Search keywords in Chinese terms were searched in the Wanfang database and China National Knowledge Internet (CNKI) from January 2000 to October 2020. 

### 2.2 Studies selection

We included RCT in which patients diagnosed with RF receive treatments. Patients were divided into the treatment group and the control group. The treatment group was given Chinese medicine for dredging collaterals (decoctions, pills, powders, Chinese patent medicines, etc.) with conventional western medicine (including low-protein diet, diuresis, blood pressure control, correction of anemia, acidosis, electrolyte disturbances, etc.). The control group was assigned conventional western medicine alone. Studies were excluded if they had less than the three endpoints mentioned below and failed to access sufficient data for pooled analysis. We excluded studies in which patients with liver fibrosis or pulmonary fibrosis may affect the RF biomarker. Moreover, conference abstract, reviews, non-randomized studies, and meta-analysis studies were excluded.

### 2.3 Endpoints

Endpoints after treatment period: (1) renal function: blood urea nitrogen (BUN) and serum creatinine (SCr) and (2) fibrosis indexes: HA, LN, type IV collagen (IV-Col), and procollagen III (PC-III) levels.

### 2.4 Data extraction

According to the inclusion and exclusion criteria, two authors (Guo and Gao) independently conducted the titles and abstracts screening of the articles after duplicated. Full-text screening and data extraction were performed in a similar manner. In the event of doubt, the articles were sent to the third author (Ding), and discussion was used when necessary to reach on consensus. Data were collected from the included articles for pooled analysis. The variables included but were not restricted to the following: title, authors, published date, number of patients included, type of interventions, and the aforementioned outcomes.

### 2.5 Quality assessment

The quality assessment of all the included RCT was carried out by means of the Cochrane Collaboration’s tool for assessing the risk of bias for Systematic Reviews of Interventions 6.0.

### 2.6 Statistical analysis

Categorical variables were calculated with the odds ratio (OR), whereas continuous variables were computed with the mean difference or standard mean difference (SMD) as effect sizes, each with 95% confidence interval (CI) results. Chi-square test was applied for heterogeneity analysis. Low heterogeneity level was indicated and fixed-effects model analysis was applied when *P* ＞ 0.10, *I* ^2^ < 50%, while low heterogeneity level was identified and random-effects model was utilized when *P* ≤ 0.10, *I* ^2^ ≥ 50%. Sensitivity analysis was tested by excluding studies with evident heterogeneity through analysis, and a fixed-effects model analysis was then applied. Small study effects and publication bias was investigated by graphical assessment of funnel plots. The Revman 5.3 Software (Review Manager Version 5.3; The Cochrane Collaboration, Copenhagen, Denmark) was applied for all statistical analyses. The level of significance was set at 0.05.


**Ethical approval:** Not applicable.
**Informed consent:** Not applicable.

## 3 Results

### 3.1 Results of included studies

**Figure 1:**
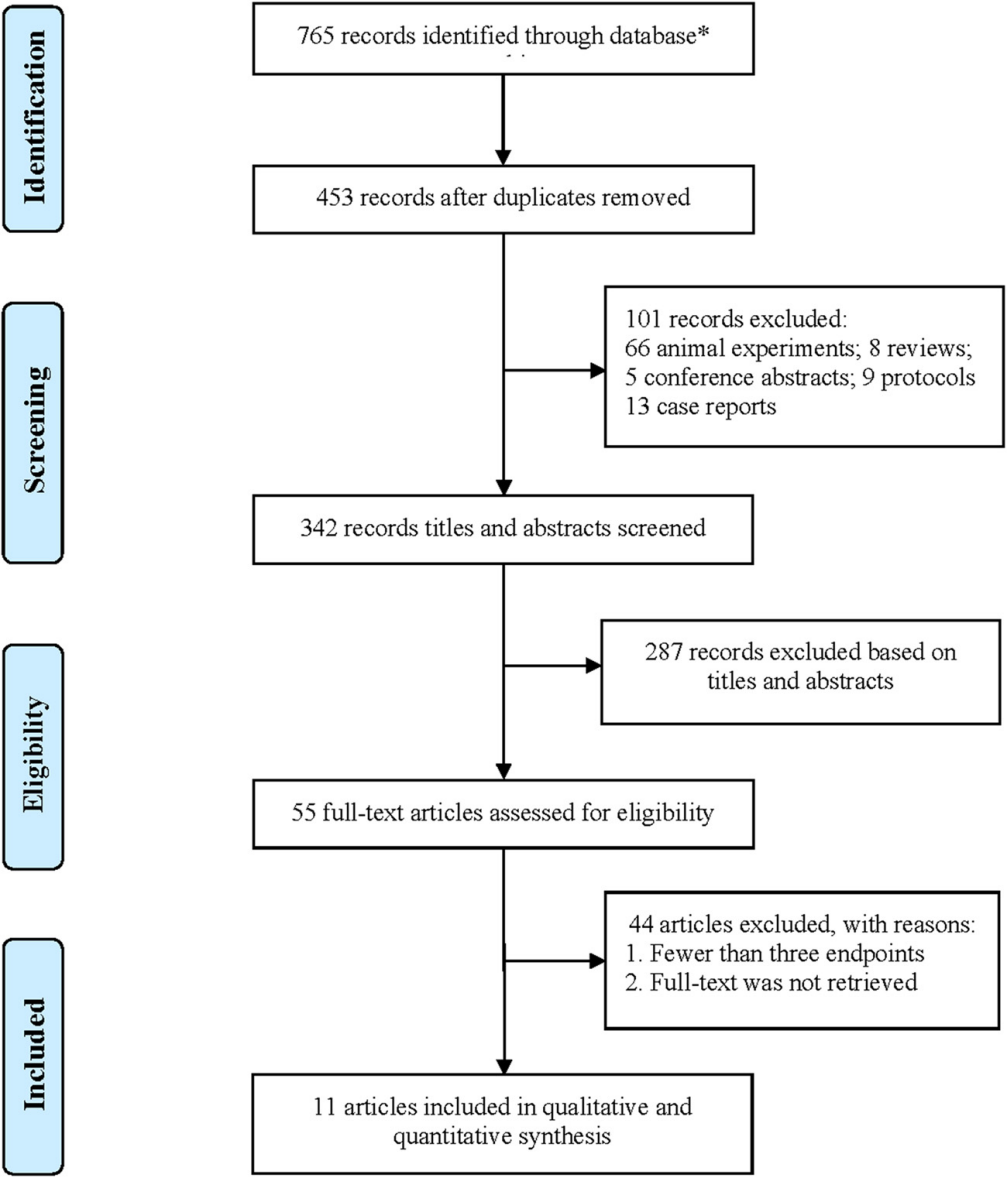
PRISMA flow diagram. *Literature search results: PubMed (18), Embase (10), Web of Science (16), The Cochrane Library (0), CNKI (589), and Wanfang Data (132).

**Figure 2:**
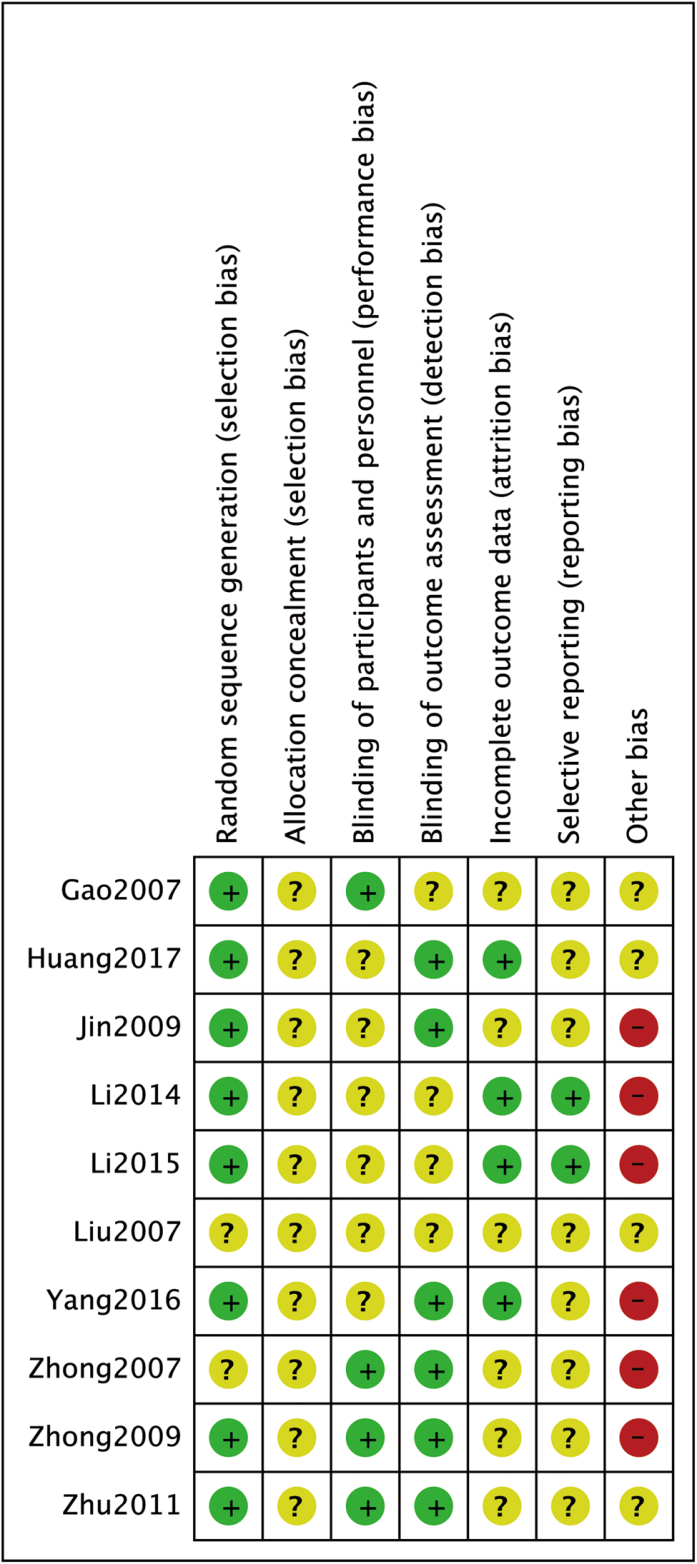
Risk of bias summary.

**Figure 3:**
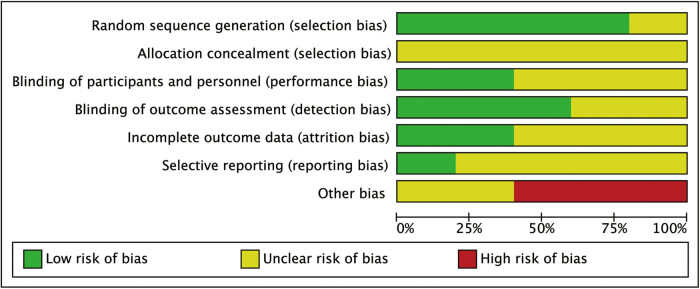
Risk of bias graph.

After the duplication of the studies derived from databases, a total of 342 articles were included for the title and abstract screening for potential full-text screening in this study. Of those, 287 articles were deemed to be irrelevant, incomplete, or describing duplicate data and were thus excluded. Eventually, eleven RCTs [15,16,17,18,19,20,21,22,23,24] were included for quantitative pooled analysis, with 898 patients. Figure 1 presents the PRISMA flow diagram. Table 1 shows the clinical characteristics of all 11 trials in which patients were allocated to the treatment group (*n* = 472) or the control group (*n* = 426). Most of the studies were inherently not blinded, attrition was not reported or unclear, and information on allocation concealment was missing. Overall, the quality of the included RCTs was low with a high risk of bias. Figures 2 and 3 illustrate the risk of bias summary and the risk of bias graph.

**Table 1:** Basic features of included studies

Included studies	Year	Cases		Age		Duration of treatment (months)	Endpoints
		(*n*)		Mean ± SD (years)			
		T1	T2	T1	T2		
Wang [12]	2017	70	70	51.2 ± 13.3	50.8 ± 12.6	4	ABCDF
Jie [13]	2015	40	40	63.8 ± 3.8	63.4 ± 3.6	1.5	ABCDEF
Jian [14]	2014	24	24	48.79 ± 10.87	49.35 ± 11.12	3	ABCDEF
Dong-Hua [15]	2016	64	64	41.3 ± 11.2	42.3 ± 10.8	3	ABCD
Li [16]	2011	35	35	40.51 ± 12.37	41.71 ± 12.41	3	ABCD
Kangkang [17]	2009	72	36	52.45	52.45	4	ABDEF
Yang [18]	2007	30	30	41.3 ± 2.2	39.8 ± 2.8	3	ADEF
Hai-Hui [19]	2007	30	30	41.27 ± 2.25	39.83 ± 2.81	2	ADEF
Yaming [20]	2008	39	39	—	—	2	ABCDF
Dan [21]	2009	38	38	40.8 ± 9.3	42.1 ± 10.6	3	DEF
Qulin [22]	2007	30	20	46.1 ± 18.2	45.7 ± 17.6	2	ABDE

T1: treatment group; T2: control group; A:BUN levels; B: SCr levels; C:HA levels; D:LN levels; E:IV-Col levels; F: PC-III levels.

### 3.2 Publication bias

**Figure 4:**
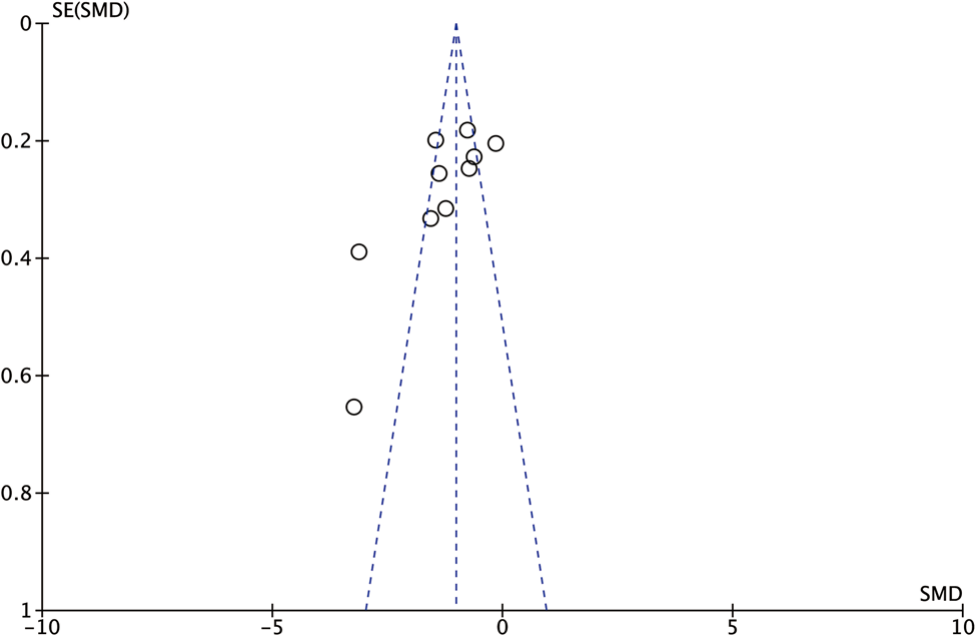
Funnel plots of the LN levels with 10 RCTs.

By visual inspection, no certain degree of asymmetry was presented in the funnel plot; hence, no publication bias was suggested for efficacy with 10 RCTs [15,16,17,18,19,20,21,23,24] (Figure 4).

### 3.3 Meta-analysis results

#### 3.3.1 BUN levels

**Figure 5:**
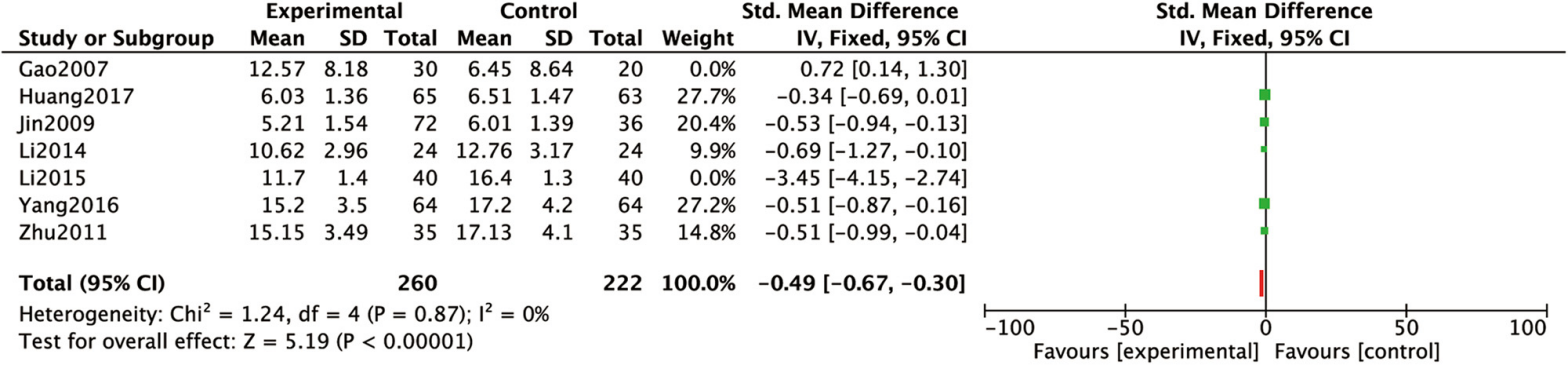
Comparison between the BUN levels of conventional western medicine with Chinese medicine for dredging collaterals and conventional western medicines alone in the treatment of RF.

The BUN levels after the treatment period were reported in seven studies [15,16,17,18,19,20,24]. Heterogeneity analysis: *P* < 0.00001, *I* ^2^ *=* 93%, indicating high heterogeneity. After removing Gao et al. [24] and Li [16] separately by sensitivity analysis, the heterogeneous results were: *P* = 0.87, *I* ^2^ = 0%, indicating low heterogeneity. Analysis results: the BUN levels of conventional western medicine with Chinese medicine for dredging collaterals in RF treatment were lower than that of conventional western medicine alone. Further RCTs or large sample is needed to make this claim (Figure 5).

#### 3.3.2 Scr levels

**Figure 6:**
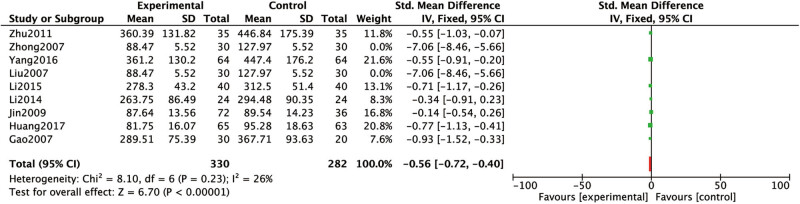
Comparison between the Scr levels of conventional western medicine with Chinese medicine for dredging collaterals and conventional western medicines alone in the treatment of RF.

The Scr levels were covered in eight studies [15,16,17,18,19,20,21,24]. Heterogeneous results: *P* < 0.00001, *I* ^2^ *=* 95%, suggesting high heterogeneity. After excluding Zhong [21] separately by sensitivity analysis, the heterogenous results were: *P* = 0.23, *I* ^2^ = 26%, demonstrating moderate heterogeneity. Analysis results: the Scr levels of conventional western medicine with Chinese medicine for dredging collaterals in RF treatment were lower than that of conventional western medicine alone (Figure 6).

#### 3.3.3 HA levels

**Figure 7:**
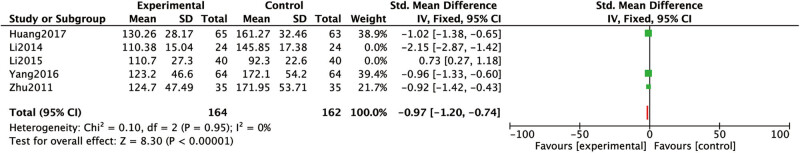
Comparison between the HA levels of conventional western medicine with Chinese medicine for dredging collaterals and conventional western medicines alone in the treatment of RF.

The HC levels were mentioned in five studies [15,16,17,18,19]. Chi-square results indicated a high heterogeneity: *P* < 0.00001, *I* ^2^ *=* 93%. After excluding Li [17] and Li [16] separately by sensitivity analysis, the heterogeneous results were: *P* = 0.95, *I* ^2^ = 0%, suggesting low heterogeneity. Analysis results: the HC levels of conventional western medicine with Chinese medicine for dredging collaterals in RF treatment were lower than that of conventional western medicine alone (Figure 7).

#### 3.3.4 LN levels

**Figure 8:**
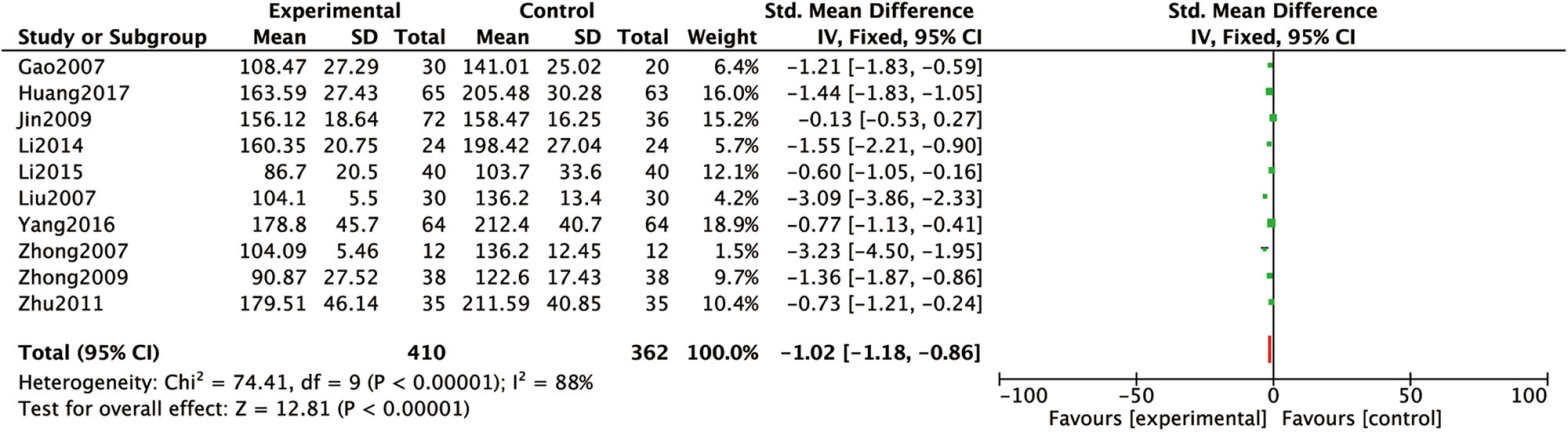
Comparison between the LN levels of conventional western medicine with Chinese medicine for dredging collaterals and conventional western medicines alone in the treatment of RF.

The LN levels were reported in nine studies [15,16,17,18,19,20,21,23,24]. Heterogeneous results: *P* < 0.00001, *I* ^2^ *=* 88%. The results demonstrated high levels of heterogeneity. Hence, a random-effects model was applied: SMD = −1.02, 95% CI (−1.18, −0.86), *P* < 0.00001 (Figure 8). Analysis results: the LN levels of conventional western medicine with Chinese medicine for dredging collaterals in RF treatment were lower than that of conventional western medicine alone.

#### 3.3.5 IV-Col levels

**Figure 9:**
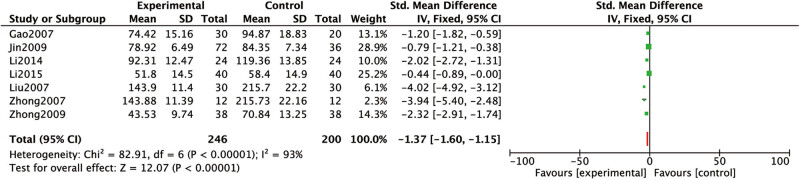
Comparison between the IV-Col levels of conventional western medicine with Chinese medicine for dredging collaterals and conventional western medicines alone in the treatment of RF.

The osteocalcin levels were mentioned in six studies [16,17,20,21,23,24]. Heterogeneous results: *P* < 0.00001, *I* ^2^ *=* 93%. The results demonstrated high levels of heterogeneity. Hence a random-effects model was applied: SMD = −1.37, 95% CI (−1.60, −1.15), *P* < 0.00001 (Figure 9). Analysis results: the IV-Col levels of conventional western medicine with Chinese medicine for dredging collaterals in RF treatment were lower than that of conventional western medicine alone.

#### 3.3.6 PC-III levels

**Figure 10:**
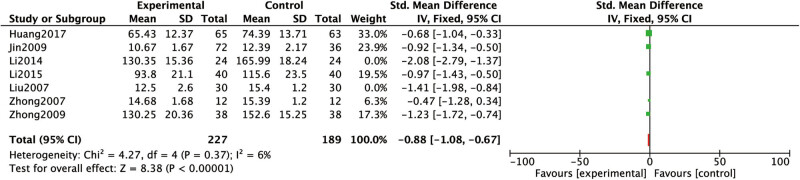
Comparison between the PC-III levels of conventional western medicine with Chinese medicine for dredging collaterals and conventional western medicines alone in the treatment of RF.

The PC-III levels were covered in six studies [15,16,17,20,21,23]. Heterogeneous results were suggested rather high: *P =* 0.01, *I* ^2^ *=* 64%. After removing Li [17] separately by sensitivity analysis, a low heterogeneity was demonstrated: *P* = 0.37, *I* ^2^ = 6%. Analysis results: the PC-III levels of conventional western medicine with Chinese medicine for dredging collaterals in RF treatment were lower than that of conventional western medicine alone (Figure 10).

## 4 Discussion

RF is a common pathway and major pathological basis for various CKD progression to end-stage renal failure [25,26]. Therefore, delaying or reversing RF is the key to treat CKD. The development of RF is generally considered to be associated with tissue damage and the response to wound healing, including a series of processes such as repair, regeneration, and tissue molding. In the pathological process of RF, normal tubular and renal interstitial structures are replaced by a large ECM. A large number of myofibroblasts appear, which eventually show progressive, irreversible damage to renal function [27].

According to the clinical characteristics of patients, RF can be attributed to the category of “edema,” “prostration closure,” and “guangge” in traditional Chinese medicine. The basic pathogenesis of RF, “deficiency and evil,” has been recognized by most contemporary physicians. “Congenital endowment deficiency,” “acquired maintenance careless,” and “yin and yang disharmony” eventually become a chronic disease. In recent years, based on the theory of collateral disease and the theory of dysentery in TCM, some scholars have proposed the “theory of dredging collaterals,” grasped the two severe conditions of “deficiency” and “blood stasis,” and formulated two basic therapeutic principles: “strengthening vital energy” and “removing blood stasis and dredging collaterals” [13]. Some animal experiments [28] have confirmed that Chinese medicine for dredging collaterals can significantly improve the endogenous hypoxic state during RIF and reduce the renal interstitium’s inflammatory response in rats to achieve the effect of delaying the progression of RF. Ba et al. [29] points that among the traditional Chinese medicine compounds that antagonize renal fibrosis, dredging collaterals are the most common. A systematic review and meta-analysis [30] have demonstrated that Chinese medicine for activating blood circulation is an effective method for treating CKD.

Scr and Bun are human metabolites, which mainly depend on renal excretion to maintain the stability of Scr and Bun content in the body. When renal function is impaired, Scr and Bun cannot be normally excreted from the body, resulting in abnormally increased Scr and Bun levels. Therefore, detecting Scr and Bun content is particularly important for judging whether renal function is impaired [31,32]. Studies have shown that Chinese medicine for dredging collaterals can regulate Scr and Bun levels in the blood and thus improve renal function but also reduce HA content and ECM deposition and inhibit RF [33]. The meta-analysis of this study showed that the BUN and Scr levels of conventional western medicine with Chinese medicine for dredging collaterals in the treatment of patients with RF were lower than that of conventional western medicine alone, indicating that Chinese medicine for dredging collaterals may be effective in improving renal function.

Studies demonstrated that the production and deposition of collagen fibers is a hallmark of RF development [34,35]. HA, LN, PC-III, and IV-Col are the main components of the ECM and better reflect the condition of renal interstitial fibrosis [36]. HA is synthesized by interstitial cells and can reflect the degree of RF activity, which is currently the best detection index in anti-fibrosis therapy. LN is the main glycoprotein in the basement membrane component, which is mainly distributed in the clear layer of the glomerular basement membrane and can interact with IV-Col to maintain the glomerular basement’s reticular structure membrane. Serum PC-III belongs to interstitial collagen that can induce fibrous crescent formation. HA, LN, PC-III, and IV-Col levels were abnormally elevated in the serum of patients with RF. In this study, the HA, LN, PC-III, and IV-Col levels of conventional western medicine with Chinese medicine for dredging collaterals in the treatment of RF were lower than that of conventional western medicine alone through meta-analysis, indicating more significant effects of fibrosis indexes.

There are several limitations to this meta-analysis. First, although the main Chinese and English databases were searched in accordance with the search strategy, the included studies are all in the Chinese population. Hence, the generality of the population may be lacking for the conclusion. Second, conventional western medicines with identical effectiveness but various components also have different effects on RF. This meta-analysis did not strictly limit components of the medicines, as well as the age, illness history, or severity of RF, resulting in bias. Finally, the results of the quality assessment indicated that further high-quality RCTs are needed to validate the conclusion.

## 5 Conclusions

In conclusion, this meta-analysis showed that Chinese medicine for dredging collaterals with lower BUN, Scr, HA, LN, PC-III, and IV-Col levels has an advantage in the treatment of RF. This finding may provide an important scientific basis for the comprehensive treatment of RF.
